# Sex Differences in the Impact of Exercise Volume on Subclinical Coronary Atherosclerosis: A Meta-Analysis

**DOI:** 10.1016/j.jacadv.2025.101786

**Published:** 2025-05-14

**Authors:** Ahmed Abdelaziz, Ahmed Elshahat, Ahmed Farid Gadelmawla, Muhammad Desouky, Abdelrahman H. Hafez, Mohamed Abdelaziz, Noha Hammad, Dua Eldosoky, Kirtipal Bhatia, Annalisa Filtz, Daniel Lorenzatti, Toshiki Kuno, Salim S. Virani, Martha Gulati, Michel D. Shapiro, Carl J. Lavie, Leandro Slipczuk

**Affiliations:** a Medical Research Group of Egypt (MRGE), Negida Academy, Arlington, Massachusetts, USA; b Faculty of Medicine, Al-Azhar University, Cairo, Egypt; c Faculty of Medicine, Menoufia University, Menoufia, Egypt; d The Brooklyn Hospital Center, Brooklyn, New York, USA; e Faculty of Medicine, Port-Said University, Port-Said, Egypt; f Faculty of Medicine, Kafrelsheikh University, Kafrelsheikh, Egypt; g Division of Cardiology, Montefiore Health System/Albert Einstein College of Medicine, Bronx, New York, USA; h Cardiology Division, Massachusetts General Hospital and Harvard Medical School, Boston, Massachusetts, USA; i Aga Khan University, Karachi, Pakistan; j The Barbra Streisand Women's Heart Center, Smidt Heart Institute, Cedars-Sinai Medical Center, Los Angeles, California, USA; k Baim Institute for Clinical Research, Boston, Massachusetts, USA; l Center for Prevention of Cardiovascular Disease, Section on Cardiovascular Medicine, Wake Forest University School of Medicine, Winston-Salem, North Carolina, USA; m Department of Cardiovascular Diseases, John Ochsner Heart and Vascular Institute, Ochsner Clinical School-the UQ School of Medicine, New Orleans, Louisiana, USA

**Keywords:** athletes, CAC, cardiovascular risks, coronary artery calcium, exercise, sex differences, subclinical atherosclerosis

## Abstract

**Background:**

The effects of high-volume exercise on coronary atherosclerosis remain controversial.

**Objectives:**

The authors aimed to evaluate the impact of endurance exercise on coronary atherosclerosis assessed by cardiac computed tomography (CT) in athletes and nonathletes, and analyze differences based on sex.

**Methods:**

We searched PubMed, Scopus, Web of Science, and Cochrane Central for relevant studies from inception to September 2024, assessing the impact of different exercise volumes on subclinical coronary artery atherosclerosis assessed by coronary artery calcification (CAC) scoring or CT angiography (CCTA). The control group comprised nonathletes. The primary outcome was the difference in CAC scores between athletes and nonathletes and the secondary outcome was the differences in calcified plaque by CCTA. The analysis was stratified by sex and exercise volume assessed using metabolic equivalents of task (MET)-min/wk.

**Results:**

Nine observational studies including 61,150 participants were included in the analysis. Male athletes with an exercise volume of >3,000 MET-min/wk showed higher mean CAC scores than nonathlete males (mean difference = 31.62; 95% CI: 10.66-52.58; *P* < 0.001), while no difference in CAC was found for male athletes with 1,500 to 3,000 MET-min/wk (*P* = 0.93) or female athletes with an exercise volume of 1,500 MET-min/wk or greater (*P* = 0.39 and *P* = 0.07). Our secondary endpoint showed significant sex-specific differences on the association of exercise volume and calcified plaque number and volume by CCTA.

**Conclusions:**

Males with high-volume exercise training (>3,000 MET-min/wk) exhibited a higher burden of calcified plaque by CAC score than male nonathletes, while no such difference was observed in female athletes.

Atherosclerosis and plaque formation are influenced by multiple factors, including sex, genetics, lifestyle, and exercise volume and duration. The relationship between endurance exercise and coronary atherosclerosis is a complex and highly debated topic in the scientific community.^1^ While regular physical activity (PA) is widely recognized for its cardiovascular (CV) benefits, including the reduction of risk factors such as hypertension, diabetes, and dyslipidemia, emerging evidence suggests that high-volume (>3,000 metabolic equivalents of task [MET]-min/wk) endurance training may paradoxically lead to a higher incidence of coronary atherosclerosis and plaque progression in athletes.^2^^,^^3^ This is particularly striking given that endurance athletes who engage in intensive PA over a long period are generally perceived to possess superior CV health compared to the general population.^4^ However, recent studies have highlighted that such athletes may be at an increased risk of developing CV disease (CVD) complications, which are typically associated with sedentary lifestyles and traditional CVD risk factors.^5^ This paradox raises important questions regarding the balance between the benefits and potential risks of extreme endurance exercise.^6^

Compelling evidence has underscored sex-specific differences in the incidence, underlying risk profiles, and clinical outcomes of CV disease.^7^ Notably, females appear to derive greater CV benefits from equivalent doses of PA compared to males,^8^ underscoring the importance of investigating study sex-specific responses to exercise in CV health. Coronary computed tomography angiography (CCTA) and coronary artery calcium (CAC) scoring are the preferred methods for the quantification and detection of subclinical coronary atherosclerosis, with CAC scoring as the standard quantification method for CAC burden, while CCTA can also visualize both calcified and noncalcified plaques.^9^

In light of these uncertainties, this systematic review and meta-analysis aimed to synthesize current evidence on the relationship between endurance exercise and coronary atherosclerosis assessed as CAC using the Agatston score or the presence of plaques on CCTA, particularly focusing on the relationship between exercise volume, CVD risk factors, and coronary atherosclerosis, stratified by sex.

## Methods

### Data sources and searches

This meta-analysis was performed according to the Preferred Reporting Items of Systematic Reviews and Meta-Analysis (PRISMA) guideline^10^ and registered in PROSPERO (International Prospective Register of Systematic Reviews) (CRD42024573617). We searched PubMed, Scopus, Web of Science, and Cochrane Library from inception until September 2024 without any language restrictions, with the following search terms: (“Athletes” OR “Runners” OR “Marathon”) AND (“Coronary atherosclerosis” OR “Coronary plaque”). The search terms, based on the retrieved databases, are presented in Supplemental Table 1. Additional manual citation analysis was adopted to search for relevant articles from previous meta-analyses and those that were not shown in the search. Two authors independently conducted the literature search, screened the titles and abstracts, and selected the final included articles. Any disagreements during the selection process were resolved by discussion and consensus with a third author.

### Study selection and outcomes

We included studies on asymptomatic individuals meeting the following inclusion criteria: 1) athletes engaged in high-volume training modalities such as running and cycling, with no prior history of coronary artery disease (CAD); 2) observational study design; 3) nonathletes who engaged in low-volume exercise and had no history of CAD; and 4) the outcome assessed in the study was subclinical coronary atherosclerosis assessed by CAC scoring or plaque evaluated by CCTA. Studies involving participants with preexisting symptomatic CVD or a history of CAD were excluded. Athletes were defined as per 2020 European Society of Cardiology guidelines as individuals who engage in regular exercise and competitive sporting events.^11^ We classified exercise volumes in athletes according to their METs relative to total exercise duration (min/wk) into 2 primary categories: 1) high volume of ≥3,000 MET-min/wk; and 2) moderate volume of 1,500 to 3,000 MET-min/wk.^11^^,^^12^ Nonathletes were defined as individuals with an exercise volume of <1,500 MET-min/wk, and no prior history of regular PA.^11^^,^^12^

The primary outcome was the CAC score assessed by a noncontrast dedicated CAC scan measured in Agatston units (AU), while the secondary outcomes were coronary plaque volume measured by CCTA,^13^ the volume of calcified plaques, and the mean number of plaques per patient assessed using CCTA. In addition, the degree of CAC (>0-10 AU, >11-100, >101-400, and >400 AU) was studied.^14^ The control group comprised nonathletes.

### Quality assessment and data extraction

Data were extracted from the included studies using prespecified extraction forms. The data consisted of the baseline characteristics of the included patients, study characteristics, and the specified outcomes. Two reviewers independently assessed the quality of the included studies using the modified Newcastle-Ottawa Scale for observational studies.^15^ The scale uses 3 main domains to assess the study quality: selection criteria, comparability testing, and outcome assessment. The risk of bias for each study was classified as good, poor, or fair. In the event of disagreement, the reviewers discussed the issues to reach consensus.

### Statistical analysis

The pooled OR for dichotomous data using event and total for each outcome, and the mean difference (MD) for continuous data using the mean and SD for each outcome, with the corresponding 95% CIs, were calculated using a random effects model. The median (IQR) were converted to mean ± SD.^16^

Heterogeneity was assessed using Cochrane's Q test and an I^2^ value ≥50% with a *P* value of ≤0.10 was considered significant heterogeneity. Separate analyses of all studied outcomes according to sex were performed. Additionally, differences in outcomes were assessed based on 2 levels of exercise volumes: 1,500 to 3,000 MET-min/wk and ≥3,000 MET-min/wk. To evaluate publication bias, the LFK plots were visually reviewed using the DOI method.

Furthermore, sensitivity analysis was conducted by excluding one study at a time (leave-one-out method) to investigate the influence of each study on the overall effect size estimate. We performed a random-effect meta-regression between mean age, baseline low-density lipoprotein cholesterol (LDL-C) levels, body mass index (BMI), and CAC scores across male and female athletes and nonathletes. All statistical analyses were performed using STATA (StataCorp LLC) 18MP.^17^

## Results

### Search results and characteristics

**Figure 1:**
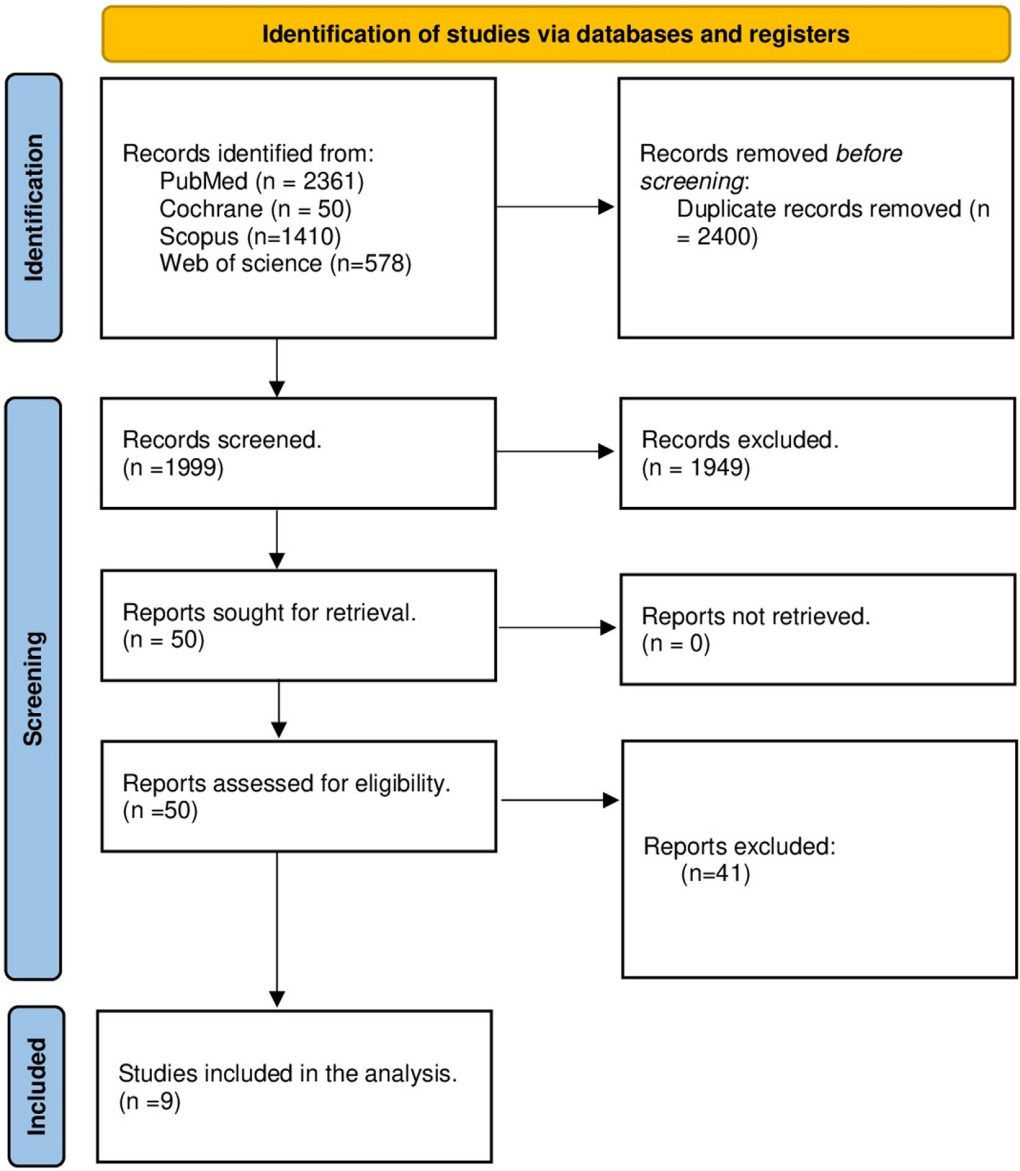
PRISMA Flow Diagram for Study Selection Process for Inclusion in the Meta-Analysis PRISMA = Preferred Reporting Items of Systematic Reviews and Meta-Analysis.

The initial literature search yielded 4,399 articles. Of these, 2,400 were duplicates and 1,999 citations were included in the title and abstract screening. The full texts of 50 articles were reviewed, and 9 studies were included in the final analysis^18^, ^19^, ^20^, ^21^, ^22^, ^23^, ^24^, ^25^, ^26^ (Figure 1).

Due to the nature of the predefined criteria, only observational studies were included in this study. The studies were conducted in several countries: 5 in the United States, one in the Netherlands, one in Belgium, one in the United Kingdom, and one in Germany. The 9 studies included a total of 61,150 participants, with 27,830 athletes and 33,320 nonathletes. Among the athletes, 8,607 (30.92%) were female, while the nonathlete group included 9,532 (28.61%) females. None of the participants had an established history of CAD, and the median age across the studies was 55 years (range: 49-61 years). Detailed summary and baseline features of the included studies are presented in Tables 1 and 2, respectively.

**Table 1:** Summary Data of Included Studies

First Author, Year	Study Arms	Site	Study Design	Medical History	Current and Former Smokers	Type of Sport	Cardiac Imaging Modality	Exposure Definition	Inclusion Criteria	Primary Endpoints	Conclusion
Aengevaeren, 2017	<1,000 MET-min/wk, 88 (30.99)	Netherlands	Retrospective cohort study	1. HTN, 7 (8) 2. DM, 1 (1) 3. FH of CHD, 29 (33)	1. Current, 7 (8) 2. Former, 32 (36) 3. Never, 49 (56)	Competitive or recreational leisure sports	CCTA scan	Individuals perform 500-1,000 MET-min/wk of exercise	1. Men ≥45 y of age 2. Asymptomatic, engaged in competitive or recreational leisure sports 3. Were free of known CVD 4. Had undergone a sports medical examination with bicycle exercise ECG that revealed no abnormalities	CAC plaques prevalence	“Participants in the >2,000 MET-min/wk group had a higher prevalence of CAC and atherosclerotic plaques. The most active group, however, had a more benign composition of plaques, with fewer mixed plaques and more often only calcified plaques. These observations may explain the increased longevity typical of endurance athletes despite the presence of more coronary atherosclerotic plaque in the most active participants”
	1,000-2,000 MET-min/wk, 121 (42.61)			1. HTN, 7 (6) 2. DM, 1 (1) 3. FH of CHD, 35 (29)	1. Current, 5 (4) 2. Former, 43 (36) 3. Never, 73 (60)			Individuals perform 1,000-2,000 MET-min/wk of exercise			
	<2,000 MET-min/wk, 75 (89.29)			1. HTN, 6 (8) 2. DM, 2 (3) 3. FH of CHD, 25 (33)	1. Current, 2 (3) 2. Former, 33 (44) 3. Never, 40 (53)			Individuals perform >2,000 MET-min/wk of exercise			
Bachman, 2020	≥3,000 MET-min/wk, 25 (58.14)	USA	Cross-sectional study	FH of CHD, 5 (20)	NR	Long-distance cycling races, ultramarathons, and Ironman triathlons	CCTA scan	Individuals who had a minimum of 10 y of cycling, running, swimming, or triathlon training and competed annually in ultraendurance events (races >6 h) such as long-distance cycling races, ultramarathons, and Ironman triathlons accounting ≥100 MET-h/wk	1. Healthy volunteers between the ages of 40 and 65 y 2. Middle-aged adults participating in ultraendurance events accounting ≥100 MET-h/wk 3. Compared to those who met current PA guidelines but were not engaged in structured endurance training accounting ≥10 MET-h/wk	1. Coronary artery calcium scores 2. Cardiac function by ECG	“Our data indicate that middle-aged ultraendurance ATH do not have marked signs of widespread cardiovascular dysfunction or elevated CHD risk compared to controls meeting physical activity guidelines”
	<1,500 MET-min/wk, 18 (41.86)			FH of CHD, 1 (6)		Jogging, walking, and recreational sports		Individuals who met current PA guidelines but were not engaged in structured endurance training accounting ≥10 MET-h/wk			
Bosscher, 2023	Lifelong Ex, 191 (34.23)	Belgium	Prospective cohort study	FH of CHD, 12 (6.3)	None	1. Cycling, 99 (51.8) 2. Running, 17 (8.9) 3. Cycling and running, 48 (25.1) 4. Duathlon, 5 (2.7) 5. Triathlon, 22 (11.5)	Dual-energy x-ray, cardiopulmonary exercise testing and CCTA scan	Athletes started regular endurance exercise training at <30 y	1. All participants were male sex and age between 45 and 70 y 2. Lifelong and late-onset athletes started regular endurance exercise training at <30 y and >30 y of age, respectively 3. Compared to nonathletes engaged ≤3 h per week in physical activity without prior exposure to regular endurance exercise	Prevalence of coronary plaques	“Lifelong endurance sport participation is not associated with a more favorable coronary plaque composition compared to a healthy lifestyle. Lifelong endurance athletes had more coronary plaques, including more noncalcified plaques in proximal segments, than fit and healthy individuals with a similarly low cardiovascular risk profile. Longitudinal research is needed to reconcile these findings with the risk of cardiovascular events at the higher end of the endurance exercise spectrum”
	Controls, 176 (31.54)			FH of CHD, 11 (6.3)		1. Cycling, 15 (8.5) 2. Running, 84 (47.7) 3. Cycling and running, 5 (2.8) 4. Nonendurance, 32 (18.2) 5. None, 40 (22.8)		Nonathletes engaged ≤3 h per week in physical activity without prior exposure to regular endurance exercise			
DeFina, 2019	<1,500 MET-min/wk, 16,447 (75.59)	USA	Prospective cohort study	ND	Current, 2,457 (14.94)	Walking, jogging or running, treadmill, bicycling, stationary cycle, swimming, aerobic dance or floor exercise, and vigorous activity	Electron beam tomography scan using the C-150XP or C-300 system (GE Imatron)	Physical activity levels of <1,500 MET-min/wk	1. All participants were male sex and age between 40 and 80 y 2. With history of physical activity and CAC scanning	1. All-cause mortality 2. CVD deaths	“This study suggests there is evidence that high levels of physical activity (3,000 MET-min/wk) are associated with prevalent CAC but are not associated with increased all-cause or CVD mortality after a decade of follow-up, even in the presence of clinically significant CAC levels”
	1,500-2,999 MET-min/wk, 3,750 (17.24)				Current, 404 (10.77)			Physical activity levels of 1,500-2,999 MET-min/wk			
	≥3,000 MET-min/wk, 1,561 (7.17)				Current, 183 (11.72)			Physical activity levels of more than 3,000 MET-min/wk			
Merghani, 2017	Athletes, 152 (62.3)	UK	Cross-sectional study	FH of CAD, 26 (17.1)	NR	Running and endurance events including marathons, half marathons, races and endurance cycling races	Transthoracic echocardiography, CCTA scan, and CMR scan	Master athletes were >40 y of age, ran ≥10 miles or cycled ≥30 miles per week and have continued to do so for ≥10 y, and competed in ≥10 endurance events, including marathons (26.2 miles, 42.2 km), half marathons (13.1 miles, 21.1 km), 10 km races, or endurance cycling races	1. Masters athletes were >40 y of age 2. Ran ≥10 miles or cycled ≥30 miles per week and have continued to do so for ≥10 y 3. Competed in ≥10 endurance events, including marathons, half marathons, races, or endurance cycling races 4. Compared to healthy controls engaged in exercise (mainly walking, jogging, or swimming)	1. Prevalence of coronary plaques 2. CAC scores	“Most lifelong masters endurance athletes with a low atherosclerotic risk profile have normal CAC scores. Male athletes are more likely to have a CAC score >300 Agatston units or coronary plaques compared with sedentary males with a similar risk profile. The significance of these observations is uncertain, but the predominantly calcific morphology of the plaques in athletes indicates potentially different pathophysiological mechanisms for plaque formation in athletic vs sedentary men. Coronary plaques are more abundant in athletes, whereas their stable nature could mitigate the risk of plaque rupture and acute myocardial infarction”
	Controls, 92 (37.7)			FH of CAD, 24 (26.1)		Mainly walking, jogging, or swimming		Healthy controls engaged in exercise (mainly walking, jogging, or swimming) in accordance with the physical activity recommendations for health			
Mohlenkamp, 2008	Marathon runners, 108 (9.09)	Germany	Prospective cohort study	HTN, 13 (12)	1. Current, 5 (4.6) 2. Former, 56 (51.9)	Marathon	Electron-beam computed tomography (CCTA) and CMR	Marathon runners	1. Males ≥50 y 2. Had completed at least 5 full-distance marathons during the preceding 3 y 3. All subjects signed an informed consent	Prevalence of coronary plaques	“Conventional cardiovascular risk stratification underestimates the CAC burden in presumably healthy marathon runners. As CAC burden and frequent marathon running seem to correlate with subclinical myocardial damage, an increased awareness of a potentially higher than anticipated coronary risk is warranted”
	Age-matched controls, 864 (72.73)			1. HTN, 353 (40.8) 2. DM, 74 (8.6)	1. Current, 245 (28.4) 2. Former, 364 (42.1)	NR		Healthy controls matched by age to marathon runners (8:1)			
	Age- and RFs-matched controls, 216 (18.18)			HTN, 61 (28.4)	1. Current, 10 (4.6) 2. Former, 112 (51.9)	NR		Healthy controls matched by age and RFs to marathon runners (2:1)			
Roberts, 2017	Marathon runners, 26 (48.15)	USA	Retrospective cohort study	1. HTN, 3 (12) 2. HLD, 6 (23) 3. FH of CAD, 24 (86) 13 (50)	Smoking Hx, 5 (20)	Marathons	Siemens Dual Source or FLASH CCTA with a minimum x-ray dose protocol	Women runners who had participated annually in the Twin Cities Marathon (Minneapolis-St. Paul, MN) for at least 10 consecutive y	1. Women runners who had participated annually in the Twin Cities Marathon for at least 10 consecutive years 2. With an average age of 56 y 3. All subjects signed an informed consent	Prevalence of coronary plaques	“Women marathon runners had minimal coronary artery calcium counts, lower coronary artery plaque prevalence, and less calcified plaque volume compared with sedentary women. Developing coronary artery plaque in long-term women marathon runners appears related to older age and more cardiac risk factors, although the runners with coronary artery plaque had accumulated significantly more years running marathons”
	Controls, 28 (51.85)			1. HTN, 16 (64) 2. HLD, 15 (60) 3. DM, 1 (4) 4. FH of CAD, 24 (86)	Smoking Hx, 15 (56)	NR		Healthy matched controls for sedentary lifestyle and age			
Schwartz, 2014	Marathon runners, 50 (68.49)	USA	Retrospective cohort study	1. HTN, 12 (25.5) 2. HLD, 22 (46.8)	Smoking Hx, 26 (52)	Marathons	Siemens Dual Source or FLASH CCTA in a minimum x-ray dose protocol	Men in the study completed at least one marathon yearly for 25 consecutive years	1. Men in the study completed at least one marathon yearly for 25 consecutive years 2. Compared to a sedentary group of men was obtained from a coronary screening study 3. With an average age of 55.3 y 4. All subjects signed an informed consent	1. Prevalence of coronary plaques 2. Total plaque volume	“Long-term male marathon runners may have paradoxically increased coronary artery plaque volume”
	Controls, 23 (31.51)			1. HTN, 15 (65.2) 2. HLD, 19 (82.6) 3. DM, 4 (17.39)	Smoking Hx, 9 (39.1)	NR		Derived from a contemporaneous CCTA database of asymptomatic healthy individuals			
Pavlovic, 2024	<1,500 MET-min/wk, 4,245 (18.15)	USA	Cross-sectional study	NR	Current smoker, 828 (19.5)	Walking, jogging, treadmill, bicycling, swimming, tennis, basketball, soccer, fitness class, water aerobics, boot camp, elliptical, rowing, jump rope, golf (without a cart), dance, stairs, hiking, and cross-country skiing	An electron beam tomography scan	Participants underwent 0 h of exercises weekly (mild physical activity intensity)	1. From 1998 to 2019 2. Men reported weekly duration of various leisure-time PAs in the prior 3 mo 3. With an average age of 51.7 y old	Prevalence of coronary plaques	“Elevated CAC was associated with lower average volume and longer duration of PA in men, providing new insight into the complex relationship between leisure-time PA behaviors and risk of CAC”
	1,500-3,000 MET-min/wk, 9,115 (38.39)				Current smoker, 1,021 (11.2)			Participants underwent weekly 2 to <5 h of exercises (vigorous physical activity intensity)			
	≥3,000 MET-min/wk, 2,227 (9.52)				Current smoker, 269 (12.1)			Participants underwent weekly ≥5 h of exercises (super vigorous physical activity intensity)			

Values are n (%).

ATH = athletes; CAC score = coronary artery calcification score; CAD = coronary artery disease; CCTA = coronary computed tomography angiography; CHD = coronary heart disease; CMR = cardiac magnetic resonance; CVD = cardiovascular disease; DM = diabetes mellitus; ECG = electrocardiogram; FH = family history; HLD = hyperlipidemia; HTN = hypertension; Hx = history; MET = metabolic equivalent of task; NR= not reported; PA = physical activity; PA = physical activity.

**Table 2:** Baseline Data of Included Patients

First Author, Year	Study Arms	Age, y	BMI, kg/m^2^	Male	Statins	Years of Exercise	Exercise Duration/wk, h	MET-Hours Per Week	Resting Heart Rate, beats/min	Systolic BP, mm Hg	Diastolic BP, mm Hg	Total Cholesterol, mg/dL	LDL Cholesterol, mg/dL	Hyperlipidemia Cholesterol, mg/dL
Aengevaeren, 2017	<1,000 MET-min/wk, 88 (30.99)	54.4 ± 6.1	25.3 ± 2.9	88 (100)	6 (7)	25.67 ± 13.1	1.43 ± 0.75	10.43 ± 5.05	NR	128 ± 11	80 ± 8	207.27 ± 33.64	NR	NR
	1,000-2,000 MET-min/wk, 121 (42.61)	54.8 ± 6.3	24.8 ± 2.8	121 (100)	2 (2)	35.67 ± 8.25	3 ± 0.9	23.93 ± 6.08		130 ± 15	80 ± 9	205.34 ± 34.03		
	<2,000 MET-min/wk, 75 (89.29)	55.9 ± 6.9	24.5 ± 2.3	75 (100)	7 (9)	40.67 ± 9.07	5.867 ± 2.041	47.467 ± 15.57		129 ± 12	80 ± 8	210.36 ± 37.12		
Bachman, 2020	≥3,000 MET-min/wk, 25 (58.14)	50 ± 1	21.6 ± 0.4	14 (56)	NR	19 ± 2	11.5 ± 0.6	123 ± 7	46 ± 1	111 ± 2	76 ± 1	204.95 ± 7.734	119.877 ± 7.734	69.61 ± 3.867
	<1,500 MET-min/wk, 18 (41.86)	49 ± 2	25.0 ± 0.9	9 (50)		NR	5.3 ± 0.5	25 ± 3	53 ± 2	110 ± 2	75 ± 2	193.35 ± 7.734	100.542 ± 7.734	77.34 ± 3.867
Bosscher, 2023	Lifelong Ex, 191 (34.23)	56 ± 7.47	23.23 ± 1.87	191 (100)	None	36.33 ± 11.2	11.67 ± 2.99	87.83 ± 21.66	54 ± 7.47	122.33 ± 12.7	75 ± 7.47	192.67 ± 29.13	121.67 ± 25.4	64.33 ± 14.19
	Controls, 176 (31.54)	55 ± 7.47	24.06 ± 2.24	176 (100)		10.33 ± 19.4	1.33 ± 2.24	14.3445 ± 10.639	61.33 ± 11.96	123 ± 11.96	75.33 ± 7.47	194.33 ± 34.38	129.67 ± 31.39	57.67 ± 12.71
DeFina, 2019	<1,500 MET-min/wk, 16,447 (75.59)	51.76 ± 8.45	28.25 ± 4.26	16,447 (100)	2,936 (17.85)	18.59 ± 13.82	1.74 ± 2.12	7.72 ± 7.83	NR	124.9 ± 14.28	NR	198.368 ± 37.68	122.72 ± 33	48.12 ± 12.375
	1,500-2,999 MET-min/wk, 3,750 (17.24)	51.167 ± 8.1	27.27 ± 3.57	3,750 (100)	589 (15.71)	23.52 ± 14.02	5.65 ± 2.84	34.67 ± 6.97		24.3 ± 14.13		195.65 ± 35.45	120.34 ± 31.23	51.867 ± 13.36
	≥3,000 MET-min/wk, 1,561 (7.17)	52.46 ± 8.56	27.1 ± 3.63	1,561 (100)	245 (15.7)	26.8 ± 14.55	12.13 ± 7.32	77.1 ± 39.43		124.64 ± 13.91		195.31 ± 35.79	119.59 ± 31.87	53.2 ± 13.7
Merghani, 2017	Athletes, 152 (62.3)	54.4 ± 8.5	NR	106 (69.74)	NR	31 ± 12.6	7.56 ± 3.54	NR	NR	125.65 ± 10.26	78.34 ± 7.63	175.56 ± 16.24	110.98 ± 14.31	NR
	Controls, 92 (37.7)	53.43 ± 7.92		54 (35.53)		NR	1.9 ± 0.34			123.58 ± 8.64	78.18 ± 6.9	171.69 ± 13.9	112.143 ± 11.52	
Mohlenkamp, 2008	Marathon runners, 108 (9.09)	57.2 ± 5.7	24.0 ± 2.3	108 (100)	NR	NR	NR	78.1 ± 38.08	65 ± 10	121 ± 14	NR	227 ± 42	121 ± 29	73.8 ± 17.3
	Age and RFs matched controls, 216 (18.18)	57.1 ± 5.6	24.9 ± 2.1	216 (100)				29.13 ± 36.67	74 ± 11	127 ± 14		215 ± 32	131 ± 31	60.6 ± 14.7
Roberts, 2017	Marathon runners, 26 (48.15)	56 ± 10	22 ± 3	0	NR	At least 10	NR	NR	57.1 ± 7.6	120 ± 13	78 ± 10	189.4 ± 31.9	103 ± 23	73 ± 15
	Controls, 28 (51.85)	61 ± 10	32 ± 8	0		NR			72.2 ± 12.1	130 ± 21	75 ± 11	198.9 ± 32.3	119 ± 36	54 ± 16
Schwartz, 2014	Marathon runners, 50 (68.49)	59.44 ± 6.66	24.16 ± 2.88	50 (100)	NR	At least 25	NR	NR	52.36 ± 9.31	127.02 ± 13.74	79.04 ± 9.4	186.44 ± 28.83	111.90 ± 26.09	58.02 ± 11.58
	Controls, 23 (31.51)	55.43 ± 10.39	30.29 ± 5.16	23 (100)					70.83 ± 10.57	134.00 ± 18.35	79.3 ± 10.39	183.56 ± 48.59	108.13 ± 45.23	46.67 ± 8.86
Pavlovic, 2024	<1,500 MET-min/wk, 4,245 (18.15)	53 ± 8.7	28.8 ± 4.6	2,491 (58.68)	786 (18.5)	NR	Zero	<25, 4,245 (100)	NR	126.1 ± 14.6	NR	200.4 ± 38	124.4 ± 33.2	47.1 ± 12.6
	1,500-3,000 MET-min/wk, 9,115 (38.39)	50.4 ± 7.9	27.5 ± 3.7	6,129 (67.24)	1,470 (16.1)		3.09 ± 0.83	1. <25, 4,867 (53.4) 2. 25-50, 2,878 (31.6) 3. ≥50, 1,370 (15)		123.1 ± 13.4		196.1 ± 36.2	121 ± 32.2	51.1 ± 13.1
	≥3,000 MET-min/wk, 2,227 (9.52)	48.3 ± 6.6	26 ± 2.9	1,991 (89.4)	252 (11.3)		8.075 ± 4.38	1. <25, 1,288 (57.8) 2. 25-50, 731 (32.8) 3. ≥50, 208 (9.3)		122.2 ± 12.9		198 ± 35.7	121.9 ± 31.6	54.2 ± 13.4

Values are n (%) or mean ± SD.

BMI = body mass index; BP = blood pressure; LDL = low-density lipoprotein; other abbreviations as in Table 1.

### Quality assessment and risk of bias

The quality assessment of the 9 included studies (Supplement Table 2) using the Newcastle-Ottawa Scale demonstrated robust community representation and appropriate ascertainment of exposure, with all studies achieving an overall rating of good quality.

### Subclinical atherosclerosis using CAC

**Figure 2:**
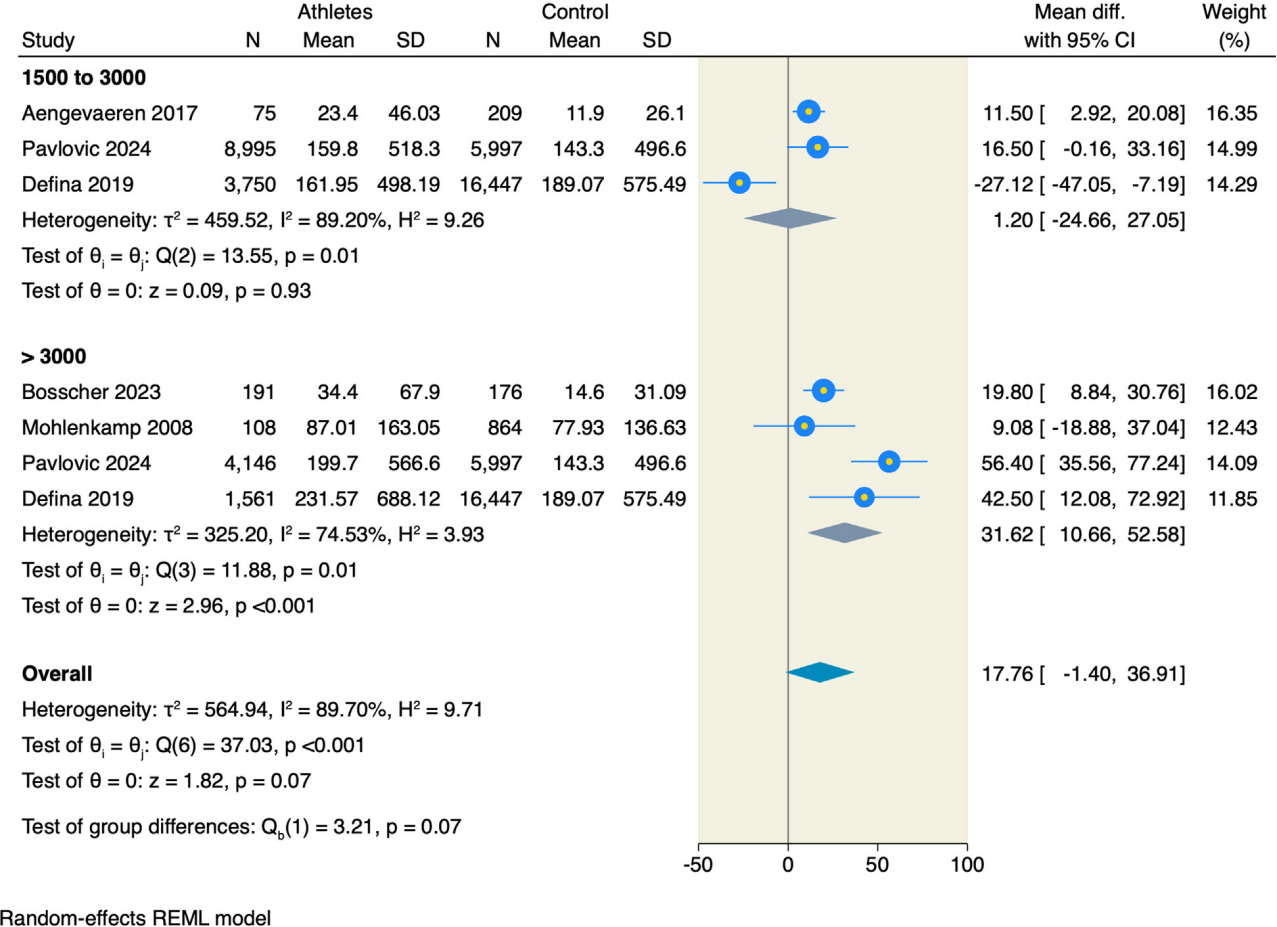
Forest Plot of CAC Score in Males With a Subgroup Analysis on Exercise Volume CAC = coronary artery calcification.

Five studies^18^^,^^20^^,^^21^^,^^23^^,^^24^ assessed CAC scores in male athletes compared to nonathletes, and the pooled estimate CAC of athletes with 1,500 to 3,000 MET-min/wk was comparable to that of nonathletes (MD = 1.2; 95% CI: −24.66 to 27.05; *P* = 0.93; I^2^: 89.2%). However, male athletes with an exercise volume of >3,000 MET-min/wk showed notably higher mean CAC scores than male nonathletes (MD = 31.62; 95% CI: 10.66-52.58; *P* < 0.001; I^2^: 74.53%) (Figure 2).

**Figure 3:**
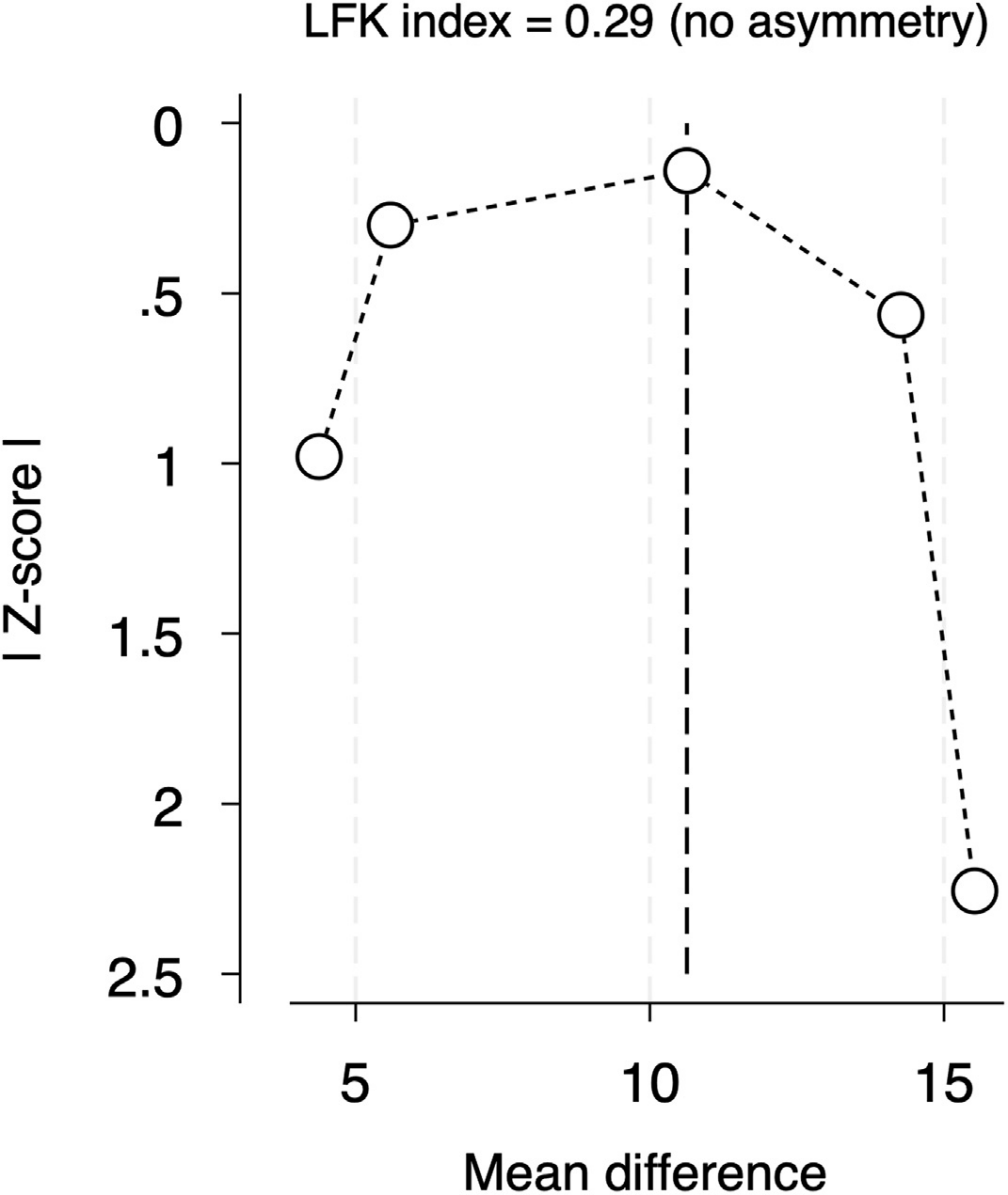
Publication Bias Assessment of CAC Score in Male Athletes and Nonathletes Using DOI Plot Method LFK index = 0.29, indicating no asymmetry is found and no publication bias is detected. Abbreviation as in Figure 2.

**Figure 4:**
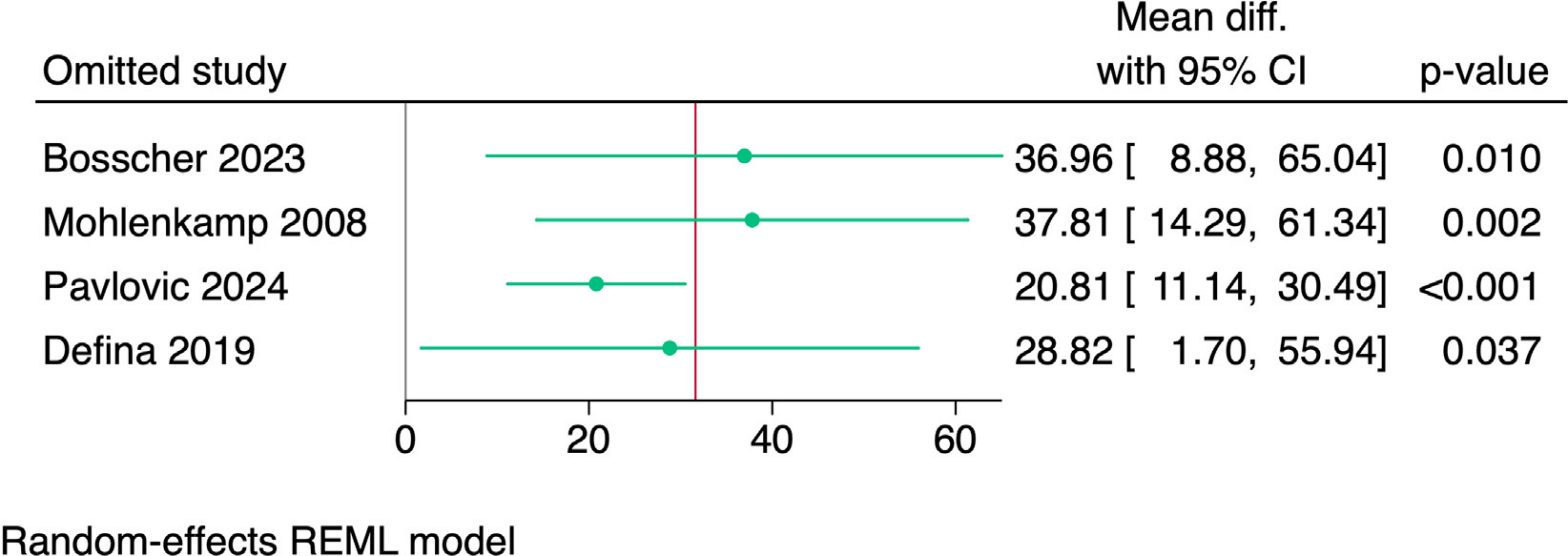
Leave-One-Out Sensitivity Analysis in Male Athletes With an Exercise Volume of >3,000 MET-min/wk MET = metabolic equivalent of task.

Publication bias was assessed using a DOI plot in which there was no asymmetry, with an LFK index of 0.29, indicating no significant publication bias (Figure 3). Moreover, we performed a sensitivity analysis (leave-one-out analysis) in athletes with an exercise volume of >3,000 MET-min/wk, and no study had a disproportional effect on the overall effect estimate (Figure 4).

**Figure 5:**
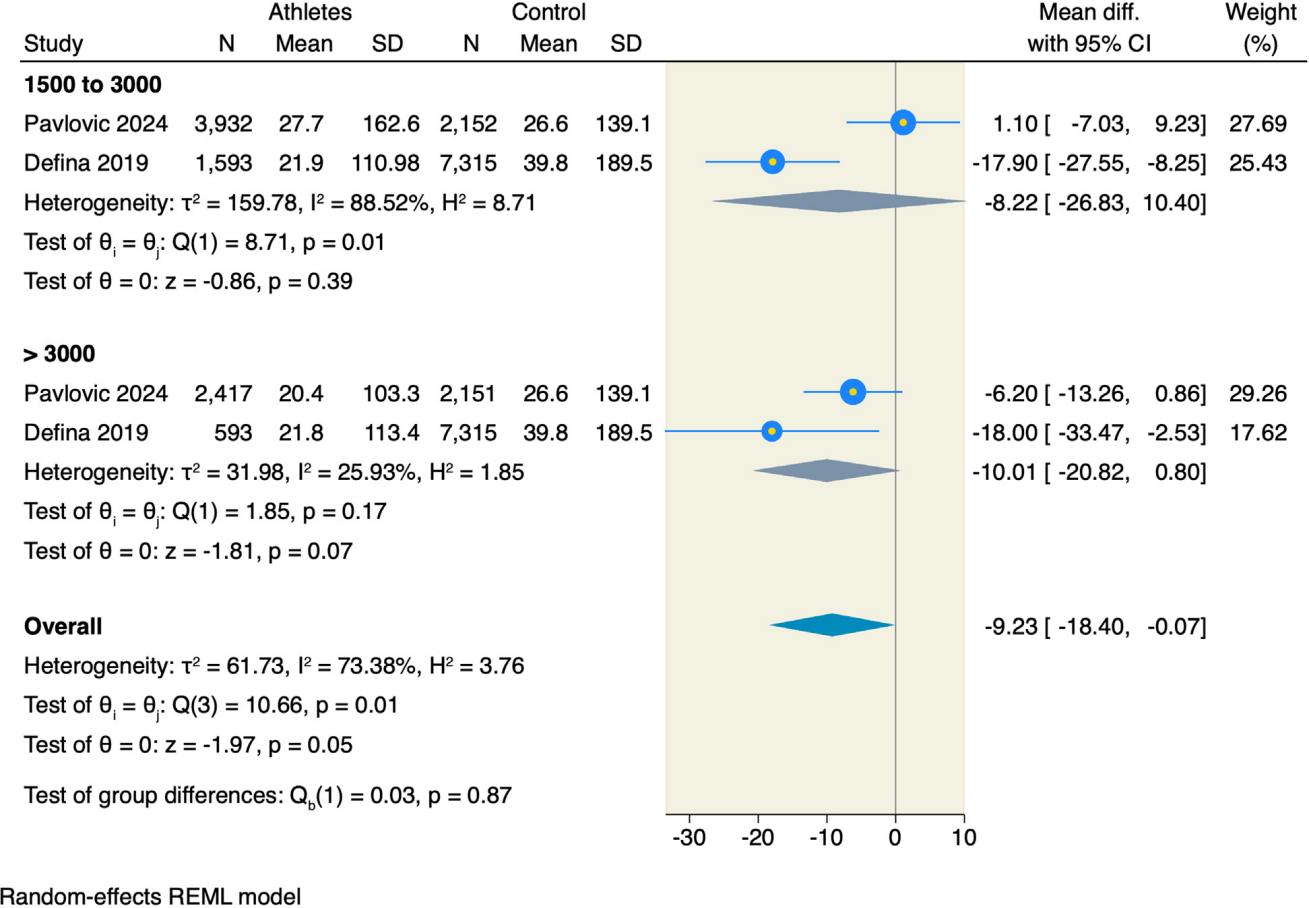
Forest Plot of CAC Score in Females With a Subgroup Analysis on Exercise Volume Abbreviation as in Figure 2.

Only 2 studies assessed the differences in CAC scores between female athletes and nonathletes. In contrast with male athletes, female athletes achieving an exercise volume of 1,500 to 3,000 and >3,000 MET-min/wk showed no statistically significant difference compared to the female nonathlete group (MD = −8.22; 95% CI: −26.83 to 10.4; *P* = 0.39; and −10.01; 95% CI: −20.82 to 0.8; *P* = 0.07) (Figure 5).

Meta-regression analysis showed no significant effect modification of mean age, baseline LDL-C levels, or BMI on the differences in CAC scores between male and female athletes and nonathletes (Supplement Table 3).

### CAC stages

The prevalence of CAC scores between 0 and 10 AU was evaluated in 4 studies of participants with exercise volumes of >3,000 MET-min/wk.^19^^,^^20^^,^^22^^,^^23^ The analysis showed no significant differences in CAC prevalence between male (OR: 1.08; 95% CI: 0.81-1.43; *P* = 0.6; I^2^ = 0%) and female athletes (OR: 0.76; 95% CI: 0.31-1.88; *P* = 0.55; I^2^ = 0%) compared with nonathletes (Supplement Figure 1). Similarly, when examining CAC scores between 10 and 100 AU, athletes (both male and female) with an exercise volume of >3,000 MET-min/wk had comparable prevalence to nonathletes, with ORs of 1.3 (95% CI: 0.74-2.3; *P* = 0.36; I^2^ = 73%) for males and 0.52 (95% CI: 0.16-1.71; *P* = 0.28; I^2^ = 0%) for females (Supplement Figure 2).

In 5 studies,^18^^,^^20^^,^^22^, ^23^, ^24^ male athletes with an exercise volume of >3,000 MET-min/wk had a higher prevalence of CAC scores between 100 and 400 AU than male nonathletes (OR: 1.41; 95% CI: 1.22-1.62; *P* < 0.001; I^2^ = 0%), without significant difference between male athletes with an exercise volume of 1,500 to 3,000 MET-min/wk and male nonathletes (OR: 1.21; 95% CI: 0.91-1.6; *P* = 0.2; I^2^ = 19.01%) (Supplement Figure 3).

Only 2 studies^22^^,^^24^ evaluated the prevalence of CAC scores >100 to 400 AU among female athletes and found no significant differences between those with exercise volumes of 1,500 to 3,000 MET-min/wk or >3,000 MET-min/wk and female nonathletes. The respective ORs were 0.98 (95% CI: 0.79-1.23; *P* = 0.89; I^2^ = 0%) and 0.87 (95% CI: 0.67-1.12; *P* = 0.27; I^2^ = 0%) (Supplement Figure 4).

Finally, for CAC scores >400 AU, 3 studies^20^^,^^22^^,^^23^ reported no significant differences between athletes with exercise volumes of >3,000 MET-min/wk and nonathletes, either for males (OR: 1.57; 95% CI: 0.94-2.61; *P* = 0.08; I^2^ = 0%) or females (OR: 0.83; 95% CI: 0.05-13.65; *P* = 0.89; I^2^ = 0%) (Supplement Figure 5).

### Subclinical atherosclerosis using CCTA

Two studies reported the mean number of calcified lesions burden per patient by CCTA, of which the pooled estimate showed that male and female athletes with an exercise volume of 1,500 to 3,000 MET-min/wk had comparable number of calcified plaques compared to the nonathletes groups with the following values (MD = −0.03; 95% CI: −0.87 to 0.81; *P* = 0.95; I^2^ = 94.87%, and −0.24; 95% CI: −0.51 to 0.04; *P* = 0.09; I^2^ = 85.63%). However, female athletes with an exercise volume of >3,000 MET-min/wk showed lower number of calcified plaques per patient compared to the female nonathletes (MD = −0.26; 95% CI: −0.42 to −0.1; *P* < 0.001; I^2^ = 0%), without significant difference in males with an exercise volume of >3,000 MET-min/wk compared to nonathletes males (MD = 0.82; 95% CI: −0.14 to 1.78; *P* = 0.09; I^2^ = 92.45%) (Table 3).

**Table 3:** Secondary Outcomes by Sex

Outcomes	Sex	Pooled Effect Sizes					
		1,500-3,000 MET-min/wk			>3,000 MET-min/wk		
		No. of Studies	Mean Difference (95% CI)	Heterogeneity (I^2^, *P* Value)	No. of Studies	Mean Difference (95% CI)	Heterogeneity (I^2^, *P* Value)
No. of calcified plaques per patients	Males	2	−0.03 (−0.87 to 0.81, *P* = 0.95)	(94.87%, *P* < 0.001)	2	0.82 (−0.14 to 1.78, *P* = 0.09)	(92.45%, *P* < 0.001)
	Females	2	−0.24 (−0.51 to 0.04, *P* = 0.09)	(85.63%, *P* < 0.001)	2	−0.26 (−0.42 to −0.1, *P* < 0.001)	(0%, *P* = 0.28)
Calcified lesions volume	Males	1	−16.23 (−27.56 to −4.9, *P* < 0.001)	–	2	26.91 (12.06-41.76, *P* < 0.001)	(0%, *P* = 0.32)
	Females	1	−10.75 (−16.52 to −4.98, *P* < 0.001)	–	2	−16.89 (−38.17 to 4.39, *P* = 0.12)	(29.18%, *P* = 0.16)
Calcified plaques	Males	1	OR 1.52 (0.96-2.42, *P* = 0.07)	–	2	OR 2.31 (0.67-7.98, *P* = 0.19)	(87.42%, *P* < 0.001)
	Females	–	–	–	–	–	–

Abbreviations as in Table 1.

Moreover, the volume of calcified plaques was lower in male and female athletes with an exercise volume of 1,500 to 3,000 MET-min/wk compared to the nonathletes' groups with the following values, respectively (MD = −16.23 mm^3^; 95% CI: −27.56 to −4.9; *P* < 0.001, and −10.75 mm^3^; 95% CI: −16.52 to −4.98; *P* < 0.001). On the other hand, the volume of calcified plaques was higher in males with an exercise volume of >3,000 MET-min/wk compared to male nonathletes (MD = 26.91 mm^3^; 95% CI: 12.06-41.76; *P* < 0.001; I^2^ = 0%), without a significant difference in the female groups (MD = −16.89 mm^3^; 95% CI: −38.17-4.39; *P* = 0.12; I^2^ = 29.18%) (Table 3).

Additionally, the prevalence of calcified plaques was reported only in male athletes. There were no significant differences observed between male athletes with an exercise volume of 1,500 to 3,000 MET-min/wk and >3,000 MET-min/wk compared to male nonathletes regarding the incidence of calcified plaques, with the following values, respectively (OR: 1.52; 95% CI: 0.96-2.42; *P* = 0.07, 2.31; 95% CI: 0.67-7.98; *P* = 0.19) (Table 3).

## Discussion

Our meta-analysis of 9 studies, encompassing 61,150 participants, addressed the effect of exercise on the prevalence of subclinical coronary atherosclerosis across different exercise volume levels (Central Illustration). The present analysis included healthy participants without any prior CVD and found the following: 1) male athletes engaging in exercise volume exceeding 3,000 MET-min/wk had significantly higher mean CAC scores and total calcified plaque volumes, as determined by CAC dedicated scans and CCTA, respectively, compared to nonathletes, while male athletes exercising between 1,500 and 3,000 MET-min/wk had significantly lower total calcified plaque volumes than nonathletes; 2) female athletes exercising above 3,000 MET-min/wk had a significantly lower number of calcified plaques per patient than nonathletes, and female athletes with exercise volumes between 1,500 and 3,000 MET-min/wk had significantly lower total calcified plaque volumes than nonathletes, as assessed by CCTA.

**Central Illustration:**
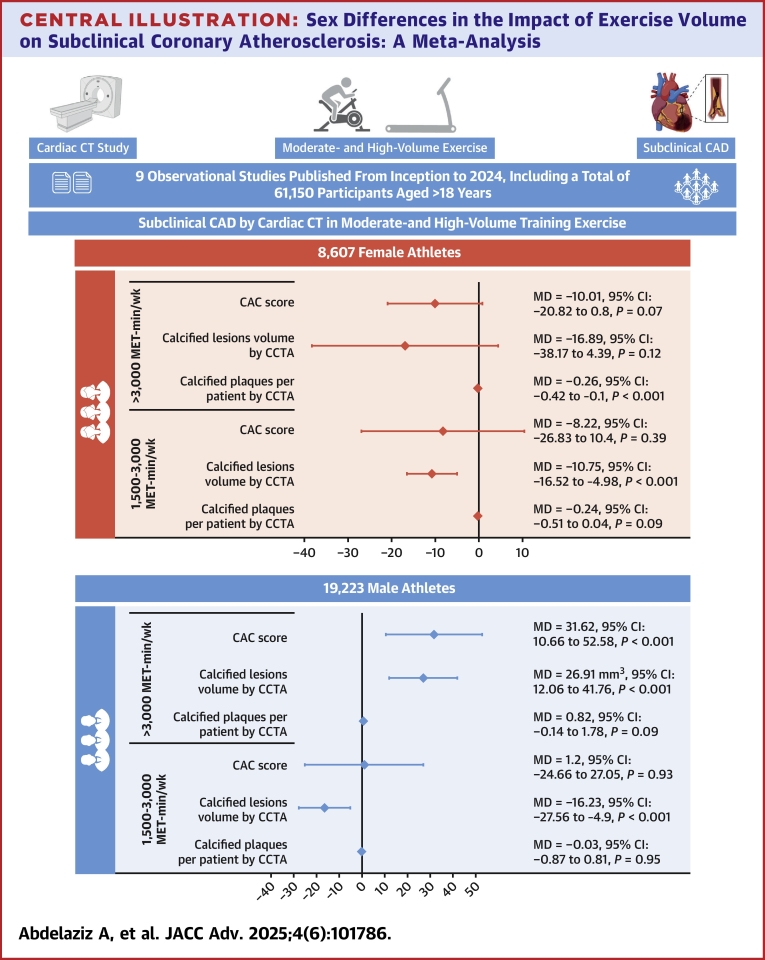
Sex Differences in the Impact of Exercise Volume on Subclinical Coronary Atherosclerosis: A Meta-Analysis CAD = coronary artery disease; CCTA = coronary computed tomography angiography; CT = computed tomography; other abbreviations as in Figures 2 and 4.

Various factors influence the development of coronary atherosclerosis in athletes; however, the precise mechanisms remain unclear. One theory suggests that elevated shear stress during intense exercise causes endothelial damage, leading to atherosclerotic plaque formation through repeated injury and calcification.^27^^,^^28^ Statin use in athletes may also contribute to plaque calcification and plaque stabilization over time. Other potential contributors include dietary intake, psychological stress, chronic inflammation, and genetics.^29^ Some studies have proposed that intense exercise may accelerate the progression of pre-existing CAD, and mechanical pressure on calcified plaques could lead to plaque rupture and thrombus formation.^30^^,^^31^ Elevated parathyroid hormone levels and inflammation during intense exercise may further promote atherosclerosis.^32^ Vascular oxidative stress, caused by intense exercise, could contribute to coronary artery calcification. Hormonal factors like testosterone may also play a role in calcification, but more research is needed.^33^^,^^34^

The influence of factors such as sex, exercise volume, ethnicity, and sport type on CAD development in athletes remains insufficiently understood. Females in particular are underrepresented in studies examining the effects of high-volume exercise on CVD outcomes. The scarcity of data in female cohorts suggests a lower prevalence of CAD among women. In a study by Merghani et al^22^ involving 244 individuals (athletes and controls) with a low 10-year Framingham Risk Score (FRS) for CAD and using CCTA, male athletes exhibited a higher likelihood of moderate to severe CAC ≥300 and atherosclerotic plaques with luminal abnormalities than controls. However, among female participants, no significant differences were observed between the athletes and controls in the number of plaques (15% vs 21%; *P* = 0.57), CAC ≥100 (7% vs 11%; *P* = 0.62), or plaque incidence. Roberts et al^26^ analyzed subclinical CAD using CCTA in 26 lifelong female marathon runners (each having completed at least one marathon annually for 10-25 years). They found these athletes had reduced coronary calcium burden, a lower prevalence of plaques, and smaller plaque volumes than controls. Our pooled analysis contrasts with previous studies which reported that high-volume female athletes had more calcified plaques per patient than nonathletes. In contrast, female athletes with moderate exercise volumes had significantly lower total calcified plaque volumes than nonathletes.^22^^,^^24^ However, the findings of other studies may be confounded by differences in baseline CVD risk profiles, as the control group had higher BMI and a greater prevalence of traditional CVD risk factors.

In most previous studies, elite male athletes showed higher CAC scores and greater coronary atherosclerosis prevalence than risk-matched controls. The Measuring Athlete’s Risk of Cardiovascular Events (MARC) study found occult CAD in 16% of 318 middle-aged athletes with low CV risk, defined as CAC >100 or significant coronary stenosis on CCTA.^35^ Mohlenkamp et al^23^ investigated subclinical atherosclerosis in 108 experienced recreational marathon runners aged 50 or older, with no history of cardiac disease, comparing them to age- and FRS-matched controls. Despite lower FRS values, 36% of runners had CAC scores >100, similar to controls. These findings are in contrast to our pooled analysis. Specifically, the referenced study found no significant differences between runners and controls regarding the prevalence of CAC scores >100. Our pooled analysis demonstrated a significantly higher prevalence of CAC scores >100 in male athletes compared to nonathletes. Potential explanations are the variability in the volume and intensity of regular PA and marathon running. Factors such as years of active running, number of marathons completed, or weekly training mileage may account for this heterogeneity, potentially affecting the paradoxical effect of excessive exercise volume on CAC scores.

The prognostic implications of high CAC scores and subclinical CAD in athletes remain unclear. Concerns have arisen that lifelong high-volume exercise may increase CV risk, as seen in a French study reporting CAD in 95% of nearly 900 sudden sports-related deaths. Athletes with high-risk pre-existing CAD may be particularly vulnerable to myocardial infarction during high-volume exertion. While calcified plaques, common in athletes with elevated CAC, are linked to reduced rupture risk, high CAC scores (>400) significantly increase the likelihood of major adverse cardiac events (MACE) compared to a CAC score of 0 (34% vs 2.1%). The Mohlenkamp trial showed a lower prevalence of zero CAC (28.7% vs 47.5%) and higher event frequency at CAC >100 compared to the MARC study.^23^

Despite elevated CAC in athletes, lower exercise levels are associated with greater atherosclerosis progression, as evidenced by the findings of Delaney et al^36^ in the MESA (Multi-Ethnic Study of Atherosclerosis) trial. Similarly, studies by Ji et al, Gao et al, and Radford et al suggest that PA mitigates CVD risks, even with high CAC scores.^8^^,^^37^^,^^38^ German et al reinforced these results, showing that increased PA reduces all-cause mortality, irrespective of CAC levels. Moreover, higher cardiorespiratory fitness (CRF) was linked to reduced CAD risk, with each 1 MET increase in CRF lowering CVD event rates by 14%.^39^ These findings underscore the protective effects of PA and CRF against CVD events, even in individuals with elevated CAC levels.^40^

The benign nature of coronary atherosclerosis in athletes has been questioned by the Master Heart study^20^ comparing 191 lifelong elite endurance athletes with 176 healthy nonathletes with low CVD risk profiles. Excluding individuals with a history of smoking, dyslipidemia, hypertension, or diabetes, the study revealed that athletes had a more significant coronary plaque burden. Athletes exhibited higher frequencies of noncalcified and mixed plaques, proximal plaques, and considerable stenosis (OR: 1.96; 95% CI: 1.24-3.11), all associated with increased CVD risk. Although CAC is linked to adverse CVD events in the general population, its implications for elite athletes remain uncertain, and elevated CAC should not discourage PA. However, it is worth noting that they used a self-report questionnaire rather than continuous tracking of training logs over time to define the different levels of exercise and atherosclerotic CVD risk factors, which may lead to underestimation of these factors at the study's baseline. Limiting their population to male participants, owing to the lower risk of CAD in females may also have a minor contribution. This study underscores the importance of integrating CT findings with CVD risk factors, symptoms, and electrocardiogram findings. This nuanced issue requires further investigation to clarify its implications for athletes' CV health.

A recent study by Pavlovic et al^24^ extended beyond conventional variables and explored the relationship between PA intensity and duration and CAC. A negative association was observed between PA intensity and CAC >100 AU, whereas the total weekly PA duration was positively correlated with CAC levels. Their study demonstrated a 2.4% increase in mean CAC per hour of PA and a 19.9% increase in mean CAC in men engaging in ≥5 hours of weekly PA vs <5 hours. They hypothesized that regular moderate-to-vigorous exercise may reduce atherogenic small, dense LDL particles, replacing them with larger, less harmful LDL. Additional benefits include reductions in blood pressure, LDL-C, triglycerides, fasting glucose, and hemoglobin A1C, alongside increases in high-density lipoprotein cholesterol. However, high PA volumes may provoke an inflammatory response, potentially contributing to plaque formation, particularly in elite athletes.

To our knowledge, this is the first meta-analysis to examine the relationship between subclinical coronary atherosclerosis and exercise volume, stratifying participants by sex and excluding those with prior CAD or CVD risk factors. Furthermore, we categorized participants based on CAC stages to approximate the extent to which CAC influences CAD risk, given that a CAC score ≥400 AU is associated with an estimated 34% risk of MACE, compared to a significantly lower risk of approximately 2.1% for a CAC score of 0.^41^^,^^42^ Additionally, we attempted to analyze CCTA data stratified by plaques per patient and calcified lesion volume, but this was feasible in only 2 studies owing to a lack of data.

### Study limitations

Several limitations may hinder the generalizability of our findings. While we attempted to stratify confounding variables, the pooled studies still offer opportunities for further validation. One such variable is race/ethnicity, as it is well established that race/ethnicity may influence CAC levels, with most studies predominantly focusing on White populations. Another major issue in the existing literature is that CAC images only calcified plaques while not visualizing noncalcified plaque. While CCTA could visualize noncalcified plaques, there was very limited data on noncalcified plaques among the included studies, precluding a separate analysis. Moreover, CCTA could also miss small amounts of CAC due to the administration of intravenous contrast, which may be important when quantifying individuals with low CAC levels. Additionally, as the studies included in our analysis were observational, they were subject to inherent biases due to the study design, and causality could not be established. Finally, there are limited data for female athletes and potential biases introduced mainly by predominantly male cohorts. These limitations underscore the need for future high-quality longitudinal studies that account for these confounding factors and establish standardized cutoff points.

## Implication on clinical practice

This meta-analysis showed a higher prevalence of CAC and plaque volume can be present in high-exercise volume male athletes, despite the lower estimated CV risk. Therefore, the possibility of subclinical coronary atherosclerosis and need for preventive therapies should not be excluded. Standard estimation of CVD risk may result in an underestimation of the actual presence of atherosclerosis in athletes. In addition, athletic conditioning, with improvements in microvascular function and vessel size among athletes, may play a role in the disassociation between subclinical coronary atherosclerosis and mortality in athletes. However, long-term assessment is mandatory to assess the relationship between subclinical atherosclerosis, exercise volume, and clinical events in athletes.

## Conclusions

Male athletes engaging in high-volume exercise had significantly higher CAC scores and total calcified plaque volume. In contrast, those with moderate-volume exercise exhibited lower plaque volumes than nonathletes. Female athletes with high-volume exercise showed lower number of calcified plaques, whereas those with moderate-volume exercise demonstrated less plaque volumes. These results highlight the relationship between exercise volumes and subclinical coronary atherosclerosis, in addition to these noted sex differences. Our findings have potential implications for individualized exercise recommendations.

However, our findings are tempered by limitations, including the observational nature of the included studies, limited data on plaque composition, and a lack of racial diversity, which restrict the generalizability and causal interpretation of the results. Future high-quality longitudinal studies are imperative to address these gaps, refine our understanding of the impact of exercise on atherosclerosis, clarify the prognostic value of subclinical coronary atherosclerosis in athletes and establish standardized thresholds for optimal exercise volume.

Perspectives **COMPETENCY IN MEDICAL KNOWLEDGE:** This meta-analysis showed that a higher prevalence of CAC and plaque can be present in high-volume male athletes, despite the lower estimated CV risk. Therefore, the possibility of subclinical coronary atherosclerosis and need for preventive lifestyle changes and therapies should not be excluded. Standard estimation of CVD risk may result in an underestimation of the actual presence of atherosclerosis in athletes. In addition, athletic conditioning, with improvements in microvascular function and vessel size among athletes, may play a role in the disassociation between CAC and mortality in athletes.

**TRANSLATIONAL OUTLOOK:** Long-term assessment is mandatory to assess the relationship between subclinical atherosclerosis, exercise volume, and clinical events in athletes.

## Funding support and author disclosures

Drs Lorenzatti, Filtz, and Slipczuk are supported by institutional grants from 10.13039/100002429Amgen and Philips. Dr Gulati has served on the advisory boards of Esperion, Novartis, and Medtronic. All other authors have reported that they have no relationships relevant to the contents of this paper to disclose.
