# Supplementary material for: Sex Differences in the Impact of Exercise Volume on Subclinical Coronary Atherosclerosis: A Meta-Analysis
Source: JACC Adv. 2025 May 14;4(6):101786. doi: 10.1016/j.jacadv.2025.101786 (PMC12144434; doi:10.1016/j.jacadv.2025.101786)
Supplement: Supplementary data [file mmc1.pdf]

Supplementay Appendix

	Page
**Supplementary Figure 1:** Forest plot of CAC >0 – 10 AU in male and female athletes with an exercise volume of > 3000 MET-min/wk.	2
**Supplementary Figure 2:** Forest plot of CAC >10 – 100 AU in male and female athletes with an exercise volume of > 3000 MET-min/wk.	3
**Supplementary Figure 3:** Forest plot of CAC >100 – 400 AU in male athletes stratified by exercise volume levels.	4
**Supplementary Figure 4:** Forest plot of CAC >100 – 400 AU in female athletes stratified by exercise volume levels.	5
**Supplementary Figure 5:** Forest plot of CAC >400 AU in male and female athletes with an exercise volume of > 3000 MET-min/wk.	6
**Supplementary Table 1:** Detailed search strategy for databases.	7
**Supplementary Table 2:** NOS tool for observational studies.	8
**Supplementary Table 3:** Meta regression analysis by sex.	9

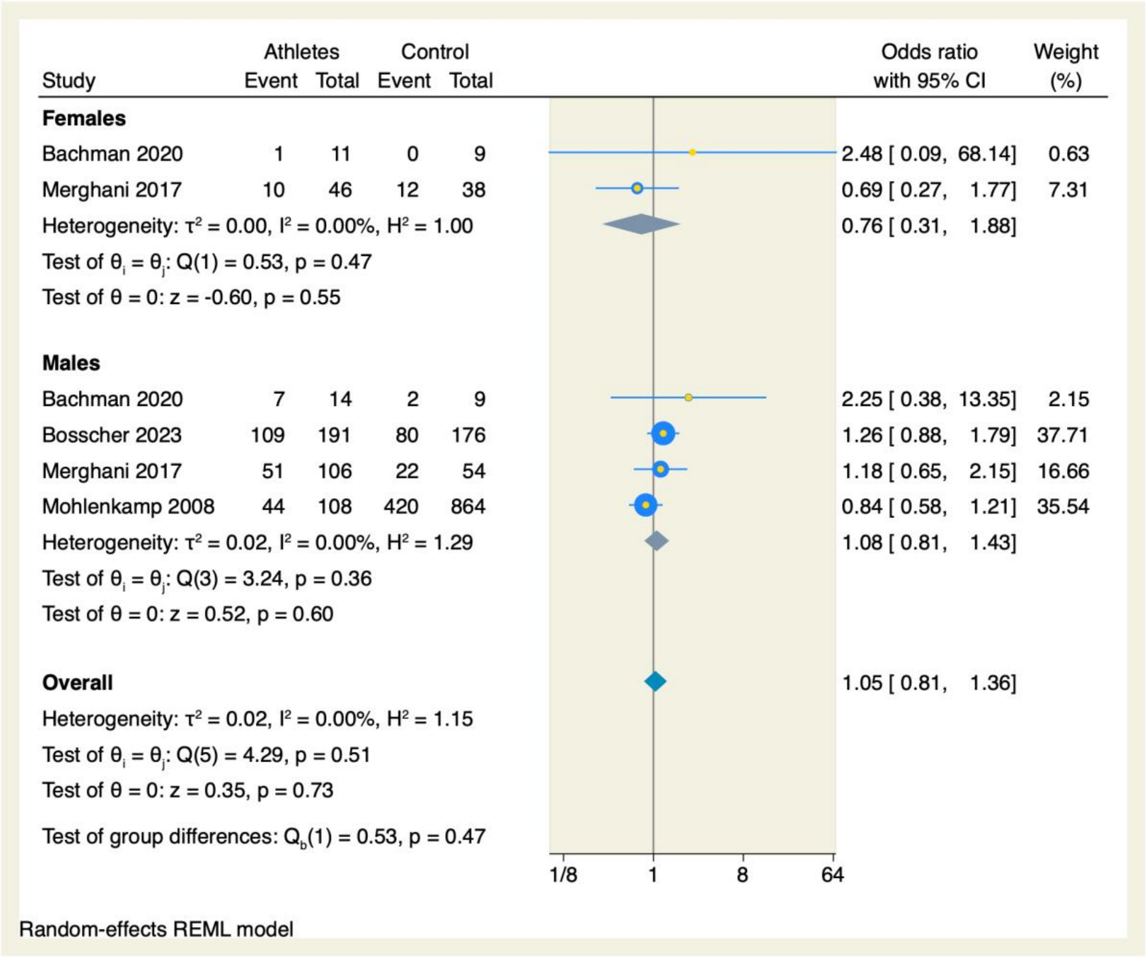

Supplementary Figure 1: Forest plot of CAC >0 – 10 AU in male and female athletes with an exercise volume of > 3000 MET-min/wk.

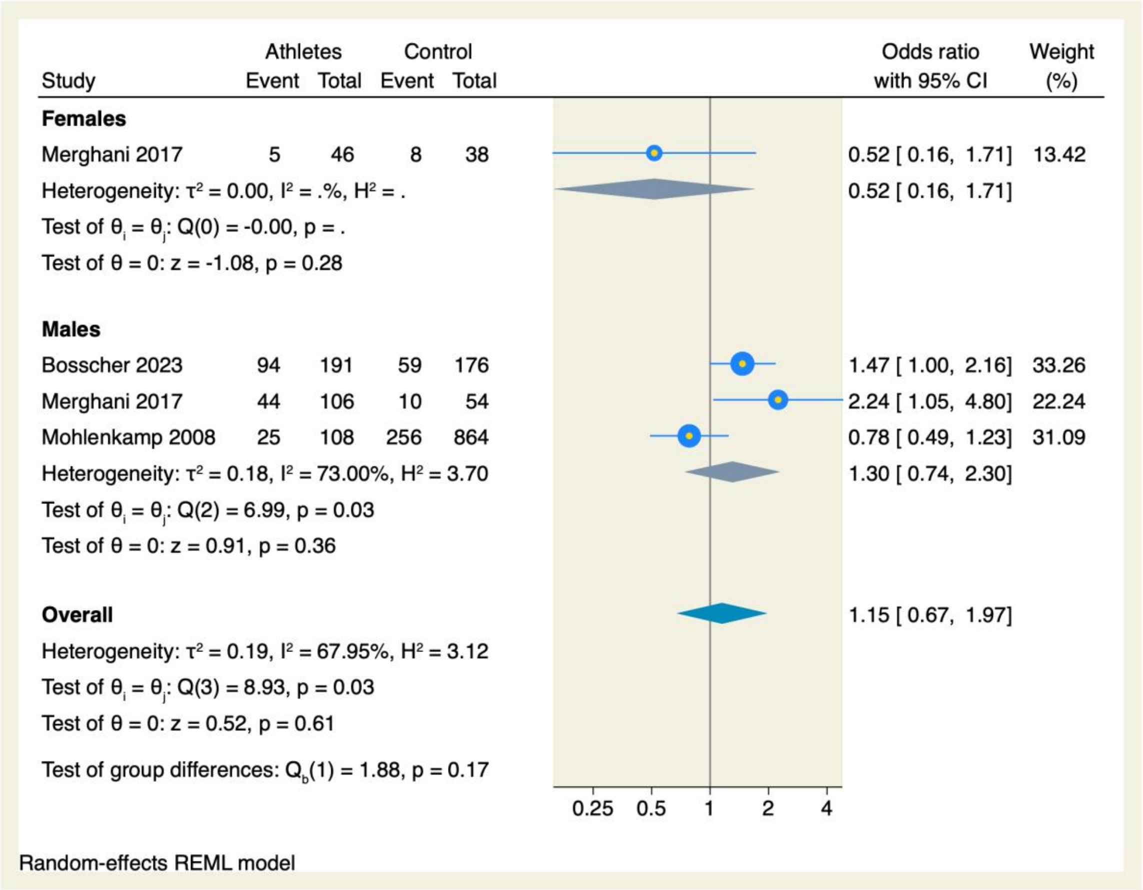

Supplementary Figure 2: Forest plot of CAC >10 – 100 AU in male and female athletes with an exercise volume of > 3000 MET-min/wk.

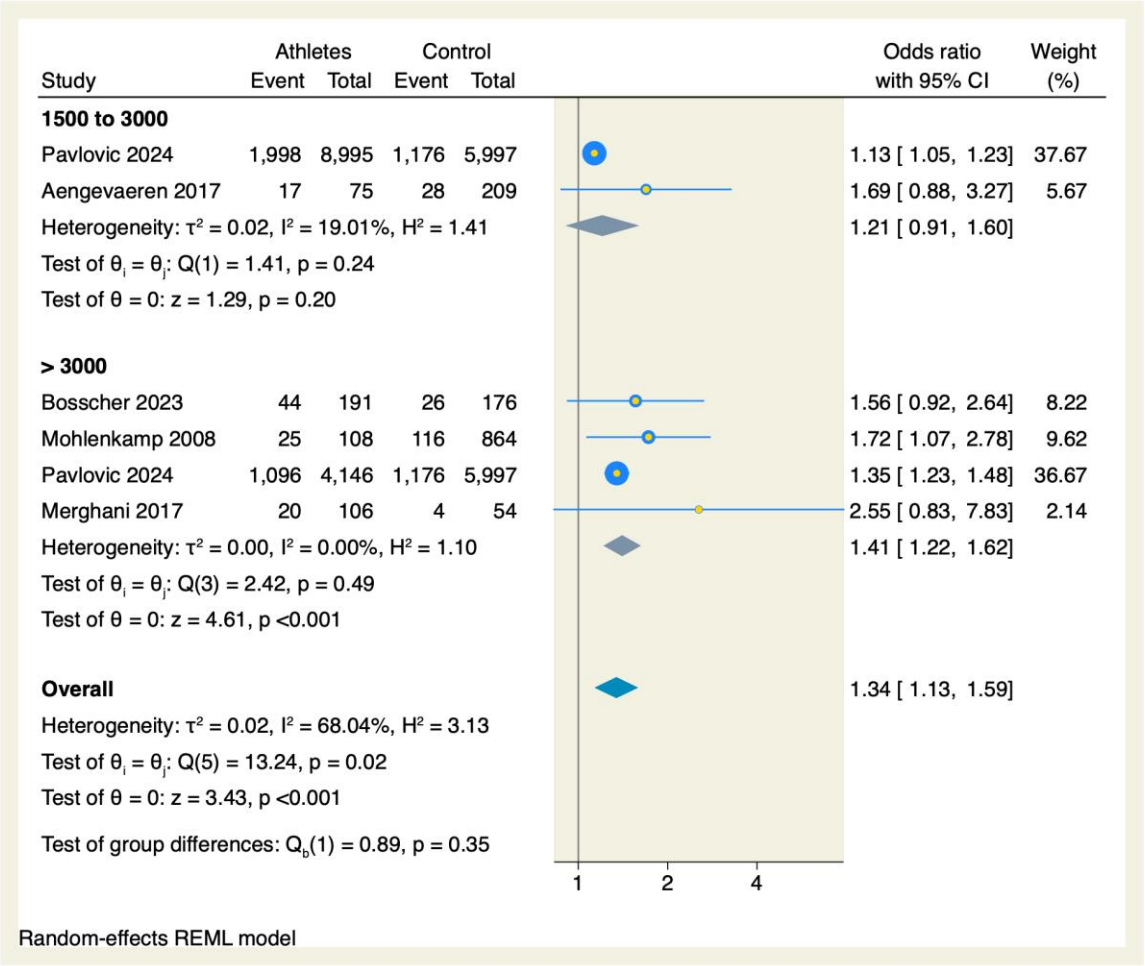

Supplementary Figure 3: Forest plot of CAC >100 – 400 AU in male athletes stratified by exercise volume levels.

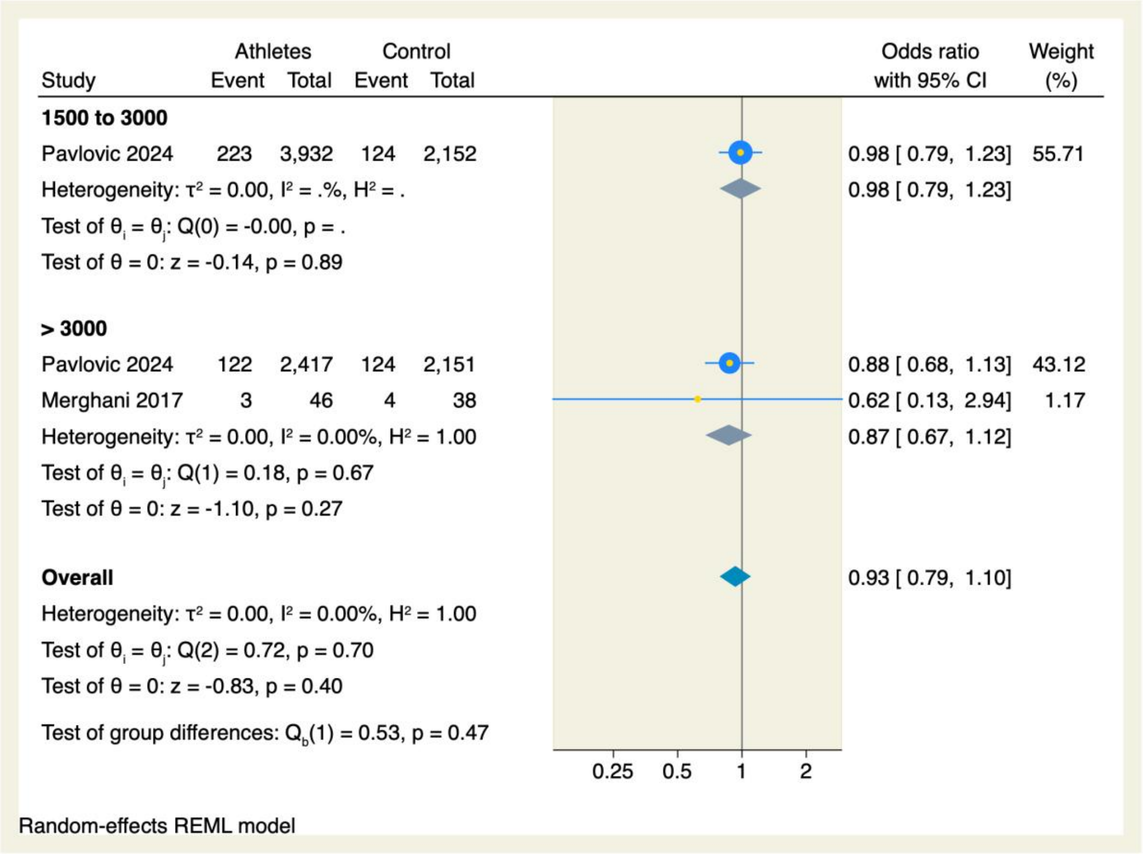

Supplementary Figure 4: Forest plot of CAC >100 – 400 AU in female athletes stratified by exercise volume levels.

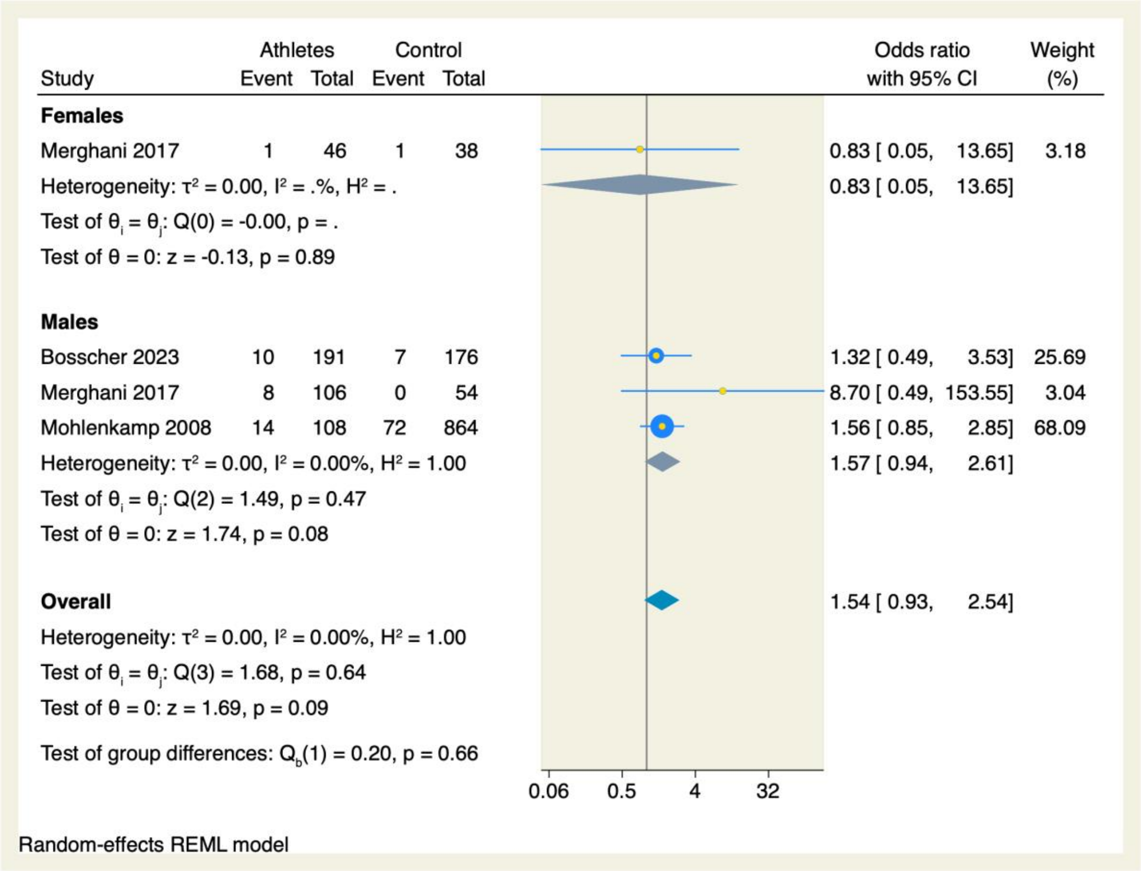

Supplementary Figure 5: Forest plot of CAC >400 AU in male and female athletes with an exercise volume of > 3000 MET-min/wk.

Appendix A: databases were used to search for articles related to the following key words:		
**Databases**	**Search Strategy**	**Results**
**PubMed**	(Athletes OR Runners OR Marathon) AND ("Coronary atherosclerosis" OR "Coronary artery calcification" OR "Cardiovascular risk" OR "Coronary plaque")	2361
**Scopus**	TITLE-ABS-KEY ((athletes OR runners OR marathon) AND (" coronary atherosclerosis" OR "coronary artery calcification" OR "cardiovascular risk" OR "coronary plaque"))	1410
**Web of Science**	(ALL=((Athletes OR Runners OR Marathon))) AND ALL=(("Coronary atherosclerosis" OR "Coronary artery calcification" OR "Cardiovascular risk" OR "Coronary plaque"))	578
**Cochrane library**	(Athletes OR Runners OR Marathon) AND ("Coronary atherosclerosis" OR "Coronary artery calcification" OR "Cardiovascular risk" OR "Coronary plaque") in Title Abstract Keyword - (Word variations have been searched)	50
**The total from the four databases:**		4399
**Number of duplicates:**		2400
**Number after removing duplication:** **(By Endnote):**		1999

Supplementary Table 1: Detailed search strategy for databases.

Study	Selection (4 stars)				Comparability (2 stars)	Outcome (3 stars)			Total stars
	Representativeness of the exposed cohort	Selection of the non-exposed cohort	Ascertainment of exposure	Demonstration that outcome of interest was not present at start of study	Comparability of cohorts on the basis of the design or analysis	Assessment of outcome	Was follow-up long enough for outcomes to occur	Adequacy of follow up of cohorts	
Bosscher 2023	*	*	*	*	**	*		*	8 stars
DeFina 2019	*	*	*	*	**	*	*	*	9 stars
Schwartz 2014	*	*	*	*	*	*	*	*	8 stars
Roberts 2017	*	*	*	*	*	*	*		7 stars
Aengevaeren (2017)	*	*	*	*	**	*	*	*	9 stars
Möhlenkamp (2008)	*	*	*	*	*	*	*		8 stars
Pavlovic 2024	*	*	*	*	**	*	*	*	9 stars
Bachman 2020	*	*	*	*	**	*	*	*	9 stars
Merghani 2017	*	*	*	*	**	*	*	*	9 stars

Supplementary Table 2: NOS tool for observational studies.

Covariates	Sex	Meta regression on CAC score			
		1500 to 3000 MET-min/week		>3000 MET-min/week	
		No. of studies	Coefficient (*p value*)	No. of studies	Coefficient (*p value*)
Age	Males	3	-3.21 (*p= 0.81)*	4	-4.95 (*p= 0.481)*
	Females	2	-4.02 (*p= 0.41)*	2	-2.60 *(p= 0.46)*
LDL	Males	3	25.22 (*p= 0.52)*	4	-5.28 (*p= 0.79)*
	Females	2	-5.71 (*p*= 0.34)	2	-7.61 (*p*= 0.41)
BMI	Males	3	-4.18 (*p*= 0.73)	4	-2.99 (*p= 0.89)*
	Females	2	-108.57 (*p= 0.34)*	2	-118 (*p= 0.42)*

Supplementary Table 3: Meta regression analysis by sex.
